# An Unexpected Evolution: Severe Pneumonia in an Immunocompromised Patient

**DOI:** 10.7759/cureus.76572

**Published:** 2024-12-29

**Authors:** Bernardo Silvério, Ana S Ramoa Oliveira, Ricardo Alves

**Affiliations:** 1 Internal Medicine, Unidade Local de Saúde do Médio Ave, Vila Nova de Famalicão, PRT; 2 Internal Medicine, Unidade Local de Saúde de Braga, Braga, PRT; 3 Critical Care, Unidade Local de Saúde de Braga, Braga, PRT

**Keywords:** immunosuppression, inflammatory bowel disease, septic shock (ss), severe community-acquired pneumonia, treatment strategies

## Abstract

Community-acquired pneumonia (CAP) varies in clinical presentation, ranging from mild pneumonia characterized by fever and productive cough to severe pneumonia characterized by respiratory distress and sepsis. We present a 40-year-old woman who presents to the emergency room with dyspnea, pleuritic chest pain, productive cough with hemoptysis, and fever. On physical examination, the patient presents with tachypnea and hypotension, which proved refractory to fluid therapy.

The analysis reveals pancytopenia and elevated C-reactive protein. The computed tomography (CT) scan shows extensive areas of consolidation and ground-glass opacities, more prominent in the right upper lung lobe. The diagnosis of septic shock with a focus on CAP was established, and the patient was admitted to the Intensive Care Unit (ICU). Later, *Streptococcus pyogenes* was identified as the causative agent of this severe pneumonia.

## Introduction

Community-acquired pneumonia (CAP) is an infection of the lung parenchyma acquired outside of the hospital, and it is a differential diagnosis for almost all respiratory diseases [1]. The clinical presentation varies from mild cases presenting with productive cough and fever to severe cases with respiratory failure and sepsis [1].

CAP is a significant cause of morbidity and mortality worldwide, and it is the leading cause of death from infectious causes [2]. In the USA, it accounts for 4.5 million emergency department admissions, and it is the second major cause of patient admission to hospitals [3]. Several risk factors predispose individuals to CAP, such as advanced age or comorbidities, such as chronic respiratory disease or conditions that confer some degree of immunosuppression [1].

The most common cause of CAP is a bacterial infection, whether by typical agents (such as *Streptococcus pneumoniae*, *Staphylococcus aureus*, or *Haemophilus influenzae*) or atypical agents (such as *Legionella* or *Mycoplasma*), and it can also be caused by viruses (such as Influenza or SARS-CoV-2). However, even after extensive research, including sputum tests and blood cultures, the etiological agent is identified in only half of CAP cases [4]. The etiology of pneumonia can be different in immunocompromised patients, as well as its progression and treatment, and this explains why these patients are often excluded from clinical guidelines [5,6].

Diagnosis is made by demonstrating infiltrates in the lung parenchyma on imaging studies in patients with suggestive clinical symptoms (such as fever, dyspnea, or productive cough), and the severity and treatment location are determined based on severity scores (such as CURB-65, which uses the presence of confusion, blood urea level, respiratory rate, systolic pressure, and age, or the Pneumonia Severity Index, which uses several variables, such as altered mental status or a history of renal or liver disease) [4]. Once again, it is important to emphasize that, due to all the mentioned specificities, in immunocompromised patients, we must consider different etiological agents and start empirical therapy with antibiotics different from the usual ones [6].

## Case presentation

We present the case of a young woman, 40 years old, a teacher by profession. She has a significant personal history of severe ileocolic Crohn's disease, treated with infliximab and azathioprine since 2018. She presented to the Emergency Department (ED) with worsening dyspnea associated with pleuritic chest pain and hemoptysis, with about 24 hours of evolution. On initial evaluation, she had a fever (auricular temperature of 39.8ºC), hypotension (blood pressure of 74/43 mmHg), tachycardia (heart rate of 123 beats per minute), and tachypnea (respiratory rate of 36 cycles per minute), despite supplemental oxygen therapy (peripheral O_2_ saturation of 90%).

**Figure 1:**
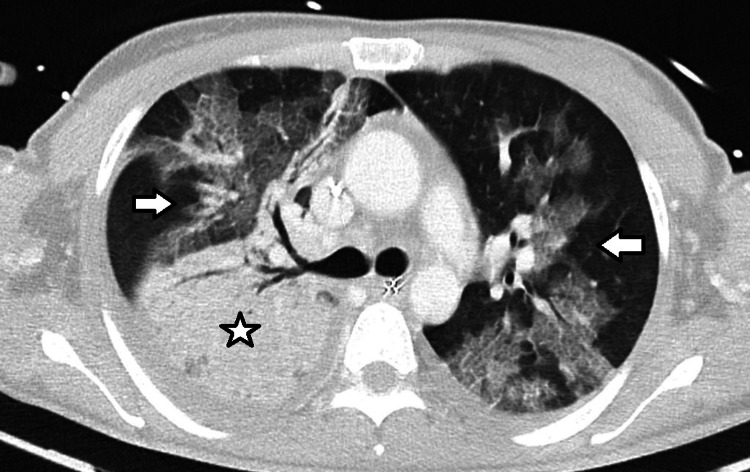
First CT scan showing extensive areas of consolidation (star) and ground-glass opacities (arrows), more pronounced in the right upper lobe, with some associated septal thickening. CT, Computed tomography

While in the ED, after fluid resuscitation with 4 L of crystalloids, she remained hypotensive and was started on norepinephrine support and hydrocortisone (50 mg every six hours). A chest computed tomography (CT) scan was performed, showing "extensive areas of consolidation and ground-glass opacities, more pronounced in the right upper lobe, with some associated septal thickening, likely of infectious nature, probably due to atypical agents. No pulmonary lobes were spared" (Figure 1).

A diagnosis of septic shock originating from bilateral CAP with multi-organ dysfunction was made, highlighting severe hypoxemic respiratory failure, hypotension with hyperlactatemia, and pancytopenia. Microbiological studies of sputum and blood cultures were collected, and empirical treatment with ceftriaxone and azithromycin was initiated. At this point, considering the state of immunosuppression and the abrupt onset of CAP with severity criteria, one might think of different agents than usual. She also started a trial of non-invasive ventilation due to respiratory fatigue and increasing oxygen requirements, and she was admitted to the Intensive Care Unit (ICU).

In the first hours after ICU admission, respiratory failure worsened, with no satisfactory response to an increased fraction of inspired oxygen (FiO_2_). It was necessary to progress to invasive mechanical ventilation (IMV), initially with an FiO_2_ of 80%. At 48 hours of ICU admission, APACHE II and SAPS II scores were calculated at 28 and 48 points, respectively, indicating an estimated mortality rate of over 50%. On the third day in the unit, *Streptococcus pyogenes* was isolated from the initial blood cultures, and, according to antibiotic sensitivity testing, the antibiotic therapy was adjusted to ceftriaxone and clindamycin (at that time, there was not enough data to suspect nosocomial infection, which is why antibiotic therapy was not escalated to a broader spectrum).

**Figure 2:**
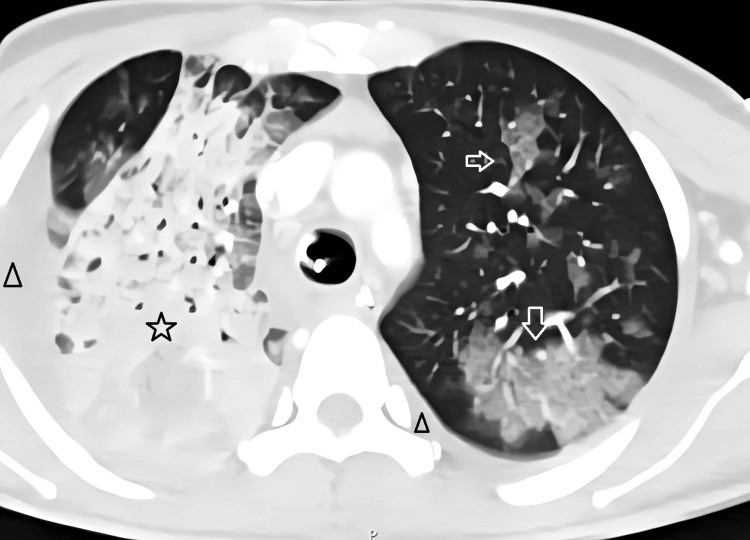
Second CT scan showing pleural effusion suggestive of bilateral empyema (triangles), extensive consolidation of the right upper lobe (star), and pronounced ground-glass opacities in various pulmonary lobes bilaterally (arrows). CT, Computed tomography

On the seventh day, there was a worsening of respiratory dysfunction and a new onset of fever. At this point, there was no longer any hematological dysfunction. We repeated the chest CT scan, which showed pleural effusion suggestive of bilateral empyema, extensive consolidation of the right upper lobe, and pronounced ground-glass opacities in various pulmonary lobes bilaterally, consistent with an extensive infectious/inflammatory process (Figure 2). A diagnostic thoracentesis was performed, yielding exudative fluid, and bronchoalveolar lavage was performed, but no microbiological agent was isolated. Nosocomial superinfection was assumed, and antibiotic therapy was escalated to piperacillin/tazobactam.

There was progressive improvement in respiratory and cardiovascular dysfunctions, leading to the discontinuation of norepinephrine support within 48 hours of the antibiotic change (the maximum dose was 10 mcg/kg/min). After 12 days of IMV (in the first five days in the ICU, it was not possible to reduce the FiO_2_ from 50%; on day 6, the FiO_2_ decreased to 45%; on days 7 and 8, when fever reappeared, the FiO_2_ was escalated to 60%; on day 9 and the following days, after changing the antibiotic for broader coverage, it was possible to progressively reduce the FiO_2_ until extubation on the 12th day), the patient was extubated, and a period of respiratory and functional rehabilitation was initiated due to significant disuse myopathy.

Given that the only identified microbiological agent in all studies, including sputum microbiology, bronchial aspirate (after intubation), bronchoalveolar lavage, pleural fluid collection, and blood cultures, was an agent that less frequently causes CAP (*S. pyogenes*), it was necessary to further detail the patient's history. It was discovered that the patient's son, a seven-month-old infant, had been diagnosed with group A *Streptococcus pharyngitis* a few days earlier, making the epidemiology of the case clearer.

## Discussion

Firstly, it is crucial to remember the importance of the patient's medical history and previous medication. In this case, the immunosuppression caused by the therapy for Crohn's disease suggested a potentially more aggressive and severe illness. It is also important to note that immunocompromised patients are susceptible to infections by less common agents, such as *S. pyogenes*. This specific agent is associated with severe infection and often causes pleural effusions and progression to septic shock, as reported by Iqbal et al. [7]. Olsen et al. [8] describe a set of characteristics of *S. pyogenes*, such as the production of extracellular proteases, various virulence factors (like the M protein), and the ability to evade the immune system, which gives it the capacity to cause invasive diseases like sepsis or septic shock.

Secondly, the significance of severity scores, combined with important clinical data such as the extent of pneumonia on imaging studies or the magnitude of infection/inflammation in lab tests, was demonstrated by this case, which led to the direct admission of a young patient to the ICU. As shown by Lisboa et al. [9], the progression of radiographic changes is a better predictor of a worse prognosis when compared to bacteremia (radiological worsening in the first 48 hours represents an independent predictor of mortality), which is exactly what happened with our patient, who presented with extensive radiological changes, worsening on the second CT scan performed. This would immediately make us think of a possible worse outcome. There are, however, other studies, such as the one conducted by Kim et al. [10], that show the presence of bacteremia in patients with severe CAP is associated with higher mortality (i.e., worse outcomes).

Thirdly, it is essential to emphasize the importance of context and epidemiological data, as these can sometimes provide clues to consider specific microbiological agents. It is well known that some changes in society's habits, such as vaccination against *S. pneumoniae*, which is used in almost every developed country, and the massive use of antibiotics, have led to changes in the main causative agents of pneumonia [11]. As we can see in this case, agents different from the usual ones, with possible resistances, are increasing, and we must be vigilant in their investigation, as they can lead to more severe courses of disease. In the case, we reported an uncommon agent, *S. pyogenes*, was identified as the cause of CAP, which supports the need to be aware of this shift in the most frequent causative agents.

As shown by Tamayo et al. [12], *S. pyogenes* is an infrequent agent causing CAP, but it is a frequent cause of CAP with severity criteria, as in the case described here. As reported by this study [12], most *S. pyogenes* infections occur in elderly patients or patients with significant comorbidities, such as, in this case, the immunosuppression caused by the treatment of Crohn's disease. Most cases of *S. pyogenes* pneumonia were community-acquired and required hospitalization for treatment [12], as in our patient. In about half of the cases, it was an invasive infection, detected in blood cultures in most of these patients [12], as we did. It is also interesting to note that, in some cases, the treatment regimen used was beta-lactam and clindamycin [12], which was our choice after isolating the agent. According to this work, there are few large-scale studies on *S. pyogenes* pneumonia, mostly case reports [12], like ours, making it difficult to establish better comparisons.

## Conclusions

We present this case to highlight several important factors to consider when approaching a patient with an established diagnosis of CAP. In this specific case, these factors include the importance of epidemiology and past history, the status of immunosuppression, the rapid unfavorable evolution, and the prompt action that led to a good outcome. It is also known that the causative agent of CAP is identified in only half of the cases, but when it is possible, adjusting antibiotic therapy can lead to a more effective fight against the infection. In this case, the identification of *S. pyogenes*, which is not a common cause of CAP, makes sense when considering the child’s pharyngitis and immunosuppression, and permitted the change of antibiotics and correct treatment of the infection.

The findings of this case can contribute to improvements in clinical practice, particularly in terms of attention to the epidemiological context and the changing agents that most frequently cause CAP today. However, it will be important to conduct new studies with larger samples to truly understand the role of *S. pyogenes* nowadays, as well as the risk factors for infection by this agent and for more severe infections.
